# Changes in the gut bacterial communities in colon cancer surgery patients: an observational study

**DOI:** 10.1186/s13099-021-00477-7

**Published:** 2022-01-04

**Authors:** Mohamed Abbas, Nadia Gaïa, Nicolas C. Buchs, Vaihere Delaune, Myriam Girard, Diego O. Andrey, Jeremy Meyer, Jacques Schrenzel, Frédéric Ris, Stephan Harbarth, Vladimir Lazarevic

**Affiliations:** 1 Infection Control Programme, Geneva University Hospitals and Faculty of Medicine, Geneva, Switzerland; 2 Genomic Research Laboratory, Division of Infectious Diseases, University Hospitals and University of Geneva, Geneva, Switzerland; 3 Division of Digestive Surgery, Geneva University Hospitals, Geneva, Switzerland; 4 Division of Infectious Diseases, Geneva University Hospitals, Geneva, Switzerland; 5 Laboratory of Bacteriology, Geneva University Hospitals, Geneva, Switzerland

**Keywords:** Gut microbiome, Colon cancer, Colon surgery, Antimicrobial prophylaxis

## Abstract

**Background:**

Colon surgery has been shown to modulate the intestinal microbiota. Our objective was to characterize these changes using state-of-the-art next generation sequencing techniques.

**Methods:**

We performed a single-centre prospective observational cohort study to evaluate the changes in the gut microbiota, i.e., taxon distribution, before and after elective oncologic colon surgery in adult patients with different antimicrobial prophylaxis regimens (standard prophylaxis with cefuroxime/metronidazole versus carbapenems for extended-spectrum beta-lactamase-producing *Enterobacterales* [ESBL-E] carriers). We obtained rectal samples on the day of surgery, intraoperative luminal samples, and rectal or stoma samples 3 days after surgery. We performed metataxonomic analysis based on sequencing of the bacterial 16S rRNA gene marker. Similarities and differences between bacterial communities were assessed using Bray–Curtis similarity, visualised using principal coordinates analysis and statistically tested by PERMANOVA. Comparison of taxa relative abundance was performed using ANCOM.

**Results:**

We included 27 patients between March 27, 2019 and September 17, 2019. The median age was 63.6 years (IQR 56.4–76.3) and 44% were females. Most (81%) patients received standard perioperative prophylaxis as they were not ESBL carriers. There was no significant association between ESBL carriage and differences in gut microbiome. We observed large and significant increases in the genus *Enterococcus* between the preoperative/intraoperative samples and the postoperative sample, mainly driven by *Enterococcus faecalis*. There were significant differences in the postoperative microbiome between patients who received standard prophylaxis and carbapenems, specifically in the family *Erysipelotrichaceae*.

**Conclusion:**

This hypothesis-generating study showed rapid changes in the rectal microbiota following colon cancer surgery.

**Supplementary Information:**

The online version contains supplementary material available at 10.1186/s13099-021-00477-7.

## Introduction

Surgical site infections (SSI) are a leading cause of healthcare-associated infections [1]. Among surgical procedures, colorectal surgery is associated with the highest incidence of SSI, despite widespread implementation of evidence-based preventive practices [2]. Indeed, in Switzerland, the average incidence of SSI in colon surgery is 14.4% [3].

Patient-level risk factors for SSI in colorectal surgery include age [4, 5], obesity [5–8], and diabetes [4]. Intraoperative risk factors include emergency surgery [9, 10], contaminated or dirty surgery [5, 10], duration of surgery, and creation of an ostomy [7, 9]; laparoscopic surgery is a protective factor [5, 11].

In the field of colorectal surgery, there is a body of indirect evidence suggesting that the microbiome plays an important role in the postoperative outcome, notably in the incidence of SSI, including anastomotic leak [12]. The current paradigm surrounding management of the high bacterial load prior to surgery is to maximise decontamination, either by perioperative antibiotic prophylaxis alone (one of the most effective measures) or in combination with bowel cleansing (mechanical bowel preparation) and/or topical oral antibiotics [13]. However, this decontamination indiscriminately affects both beneficial and potentially pathogenic bacteria.

There is a real gap in the literature regarding adequate description of the modulation in the intestinal microbiota in patients undergoing colon surgery using state-of-the-art next-generation sequencing techniques.

Therefore, we performed a prospective observational study to evaluate changes in the gut microbiota in adult patients undergoing perioperative antibiotic prophylaxis regimens and elective colon surgery, and to assess differences between preoperative (rectal) and intraoperative (luminal) microbiome. Because all patients received perioperative antibiotic prophylaxis, we believe that the changes in the gut microbiota would be due to the impact of antibiotics.

## Methods

We performed a single-centre prospective observational cohort study including adult patients who underwent elective colon surgery at Geneva University Hospitals, Switzerland, which is a regional tertiary centre.

Eligible patients were identified from the operating programme and were included if they were adults (age ≥ 18 years), and were scheduled to undergo elective colon surgery for oncological reasons. Informed consent was obtained during the preoperative surgical visit. Included patients have also been enrolled in a prospective cohort in a national surveillance system of SSI (Swissnoso) [3]. Exclusion criteria were emergency colon surgery, rectal surgery, receiving any topical oral or systemic oral/parenteral antibiotic within 30 days preceding the colon surgery, and necessity of mechanical bowel preparation. Patients who were unable or unwilling to provide informed consent or for whom 30-day follow-up was difficult/impossible (e.g., residing overseas) were also excluded.

We collected patient data on their digestive colonisation status with extended-spectrum beta-lactamase producing *Enterobacterales* (ESBL-E) as well as demographic and medical data, from the electronic medical health record.

According to institutional perioperative prophylaxis guidelines, patients receive a second-generation cephalosporin (cefuroxime) plus metronidazole within 60 min preceding the incision, except those known to have intestinal colonisation with ESBL-E who receive ertapenem, as non-targeted prophylaxis is associated with an increased risk of SSI [14].

Patients in our study were all included in our institutional Fast-track protocol [15]. Briefly, nutritional status was assessed one month prior to surgery. In case of impaired nutritional status (as testified by low serum pre-albumin levels and a Nutritional Risk Score (NRS) ≥ 3 [16], patients received protein-enriched oral supplements. No dietary restrictions were enforced before surgery. Non-diabetic patients were given a hyperglycaemic oral solution (ProvideXtra®, Fresenius Kabi, Germany) the night before (400 ml) and two hours (200 ml) prior to surgery. A low-fibre diet was introduced at postoperative day 1, and was continued for approximately two weeks. Protein-enriched oral supplements were given between postoperative days 2 and 7.

### Sample collection

Three samples were collected from included patients on two occasions using Copan ESwab (480 CFA, Regular Flocked Swab with Liquid Amies Medium). The first sample was a rectal swab collected on the day of surgery, just after the patient had undergone general anaesthesia. The second was a swab of luminal stool in the proximal colonic divided segment. The third sample was a rectal (or stoma) swab performed on postoperative day 3. The samples were immediately frozen at –80 °C, pending batched metataxonomic analysis.

### DNA extraction, 16S rRNA gene amplification and sequencing

Thawed ESwab was homogenised by vortexing during 1 min. DNA was extracted from 250μL of suspension using ZymoBIOMICS DNA Miniprep kit (Zymo Research, US) and eluted in 60μL of H_2_O. Purified DNA was quantified using the Qubit dsDNA BR Assay Kit (Thermo Fisher Scientific, US) according to manufacturer’s instructions and stored at − 20 °C. Three no-sample controls were performed by extracting DNA using the same extraction procedure but omitting the addition of a sample.

The V3–4 region of the bacterial 16S rRNA genes was amplified using 1 ng of extracted DNA or 6µL of the extract of no-sample controls, as previously described [17].

The sequencing library construction using the MetaFast protocol, Illumina MiSeq 2 × 300 sequencing with MiSeq Reagent Kit v3 and initial sequence processing (demultiplexing and removal of adapter and primer sequences) were performed externally at Fasteris (Plan-les-Ouates, Switzerland).

### Bioinformatics

Paired reads were quality filtered and joined using PEAR v0.9.11 (-m 470 -n 390 -t 150 -v 10 -q 33 -p 0.0001 -u 0) [18]. Merged sequence reads were clustered into zero-radius operational taxonomic units (zOTUs) using UNOISE3 [19] from the USEARCH v11.0.667 package [20].

From the sample dataset, we removed zOTUs matching any of the following criteria: (i) presented < 90% identity to reference bacterial sequences in the EzBioCloud 16S database [21] (downloaded on the 19^th^ of August 2019) as revealed by USEARCH [22] (-id 0.90 -query_cov 0.99); (ii) were represented by ≤ 10 counts; and (iii) had higher relative abundance in no-sample controls than in clinical samples. zOTUs were classified using EzBioCloud 16S database via MOTHUR v1.43.0 [23] using command classify.seqs (method = wang cutoff = 80). Sequencing data were submitted to the European Nucleotide Archive (ENA; www.ebi.ac.uk/ena; study number: PRJEB44214).

### Bacterial community comparisons

Bacterial communities were clustered using Bray–Curtis similarity [24] matrix constructed in PRIMER v7 (PRIMER-E Ltd, Plymouth, UK) based on square root-transformed relative abundance of zOTUs. Similarities and differences between communities were visualised using principal coordinates analysis (PCoA). To assess significance of differences in overall microbiota taxonomic composition between groups defined by categorical variables, we used permutational multivariate analysis of variance [25] (PERMANOVA) test (*adonis2* function in vegan v2.5–7 R v3.6.1 package) with 9,999 permutations. The homogeneity of multivariate dispersion (average distance to the group centroid) was assessed by PERMDISP [26] using *betadisper* and *permutest* (with 9,999 permutations) functions in vegan [27]. To analyse the relationship between bacterial community profiles and quantitative variables, we used a distance-based linear model (DISTLM, PRIMER) with 9,999 permutations.

To identify differentially abundant taxa (from phylum down to the species level), we used the analysis of composition of microbiomes (ANCOM) [28] with the following settings: adjusted = F, repeated = F, multcorr = 2 (“less stringent” multiple comparison correction), sig = 0.05, prev.cut = 0.75 (features not observed in ≥ 75% samples were omitted) and, when appropriate, paired.test_ancom = paired. The results that passed the 0.6 threshold were considered significant.

The Shannon diversity index was calculated from the relative abundance of zOTUs in PRIMER.

### Ethical approval

The study was approved by the local Ethics Committee (Geneva, no. 2018-02379) and complied with the declaration of Helsinki.

## Results

Between March 27, 2019 and September 17, 2019 28 adult patients were scheduled to undergo elective colorectal cancer surgery and were therefore considered as eligible for the study. One patient was excluded because the postoperative sample was not collected, leaving 27 patients in the final analysis. The median age was 63.6 years and 44% were female. One patient was operated for a non-resectable polyp, two were operated for neuroendocrine tumours, and the remaining 24 patients had adenocarcinoma. Table 1 summarises other patient characteristics.

**Table 1:** Characteristics of patients

Characteristic	Frequency (%) or median (IQR)
Age (years)	63.6 (56.4–76.3)
Gender (female)	12 (44.4)
Body mass index (kg/m^2^)	25.0 (22.6–27.3)
Prealbumin^a^ (mg/l)	270 (232–306)
Preoperative blood glucose (mmol/l)	5.7 (5.3–6.9)
Preoperative haemoglobin (g/l)	133 (113–145)
Postoperative haemoglobin, day 3 (g/l)	118 (105–122)
MDR-GNB intestinal carriage^b^	
None	20 (74.7)
ESBL	3 (11.1)
CRE and ESBL	1 (3.7)
Type of tumour	
Adenocarcinoma	24 (88.9)
Neuroendocrine tumour	2 (7.4)
Non-resectable polyp	1 (3.7)
Tumour location	
Ascending colon	12 (44.4)
Transverse colon	2 (7.4)
Descending colon	7 (25.9)
Sigmoid colon	6 (22.2)
TNM stage*^c^	
T1	2 (7.4)
T2	6 (22.2)
T3	9 (33.3)
T4	6 (22.2)

*CRE* carbapenem-resistant Enterobacteriaceae, *ESBL* extended-spectrum beta-lactamase, *IQR* interquartile range, *MDR-GNB* multi-drug resistant Gram-negative bacteria

^*^ for adenocarcinoma patients only (n = 24)

^a^ 1 missing value

^b^ 3 missing values

^c^ 1 missing value

Most (81.5%) patients received standard perioperative antimicrobial prophylaxis as they were not ESBL-E carriers (Table 2). Four patients (14.8%) received ertapenem due to ESBL-E carriage, although 1 patient was subsequently found to have a negative screening swab. One patient received meropenem due to a history of carriage of carbapenem resistant *Enterobacterales* (*Escherichia coli* harbouring the OXA-48 gene, phenotypically susceptible to meropenem). Two patients developed SSI: one patient who received ertapenem for ESBL *E. coli* carriage developed superficial SSI with *Streptococcus anginosus*, *Staphylococcus lugdunensis*, and *Enterococcus faecalis*, as revealed by routine culture; another patient, who received standard prophylaxis, developed organ/space SSI and the recovered intraoperative microorganisms were *S. anginosus*, *E. faecalis*, and *Colinsella aerofaciens*, as revealed by routine culture.

**Table 2:** Operative characteristics

Characteristic	Frequency (%) or median (IQR)
Perioperative antimicrobial prophylaxis	
Cefuroxime + metronidazole	22 (81.5)
Ertapenem	4 (14.8)
Meropenem*	1 (3.7)
Laparoscopically-assisted surgery	
Yes	21 (77.8)
No	3 (11.1)
Conversion to laparotomy	3 (11.1)
Duration of procedure (minutes)	190 (163–243)
Restoration of bowel continuity	22 (81.5)
Intraoperative complication	1 (3.7)
Surgical site infection	
Superficial	1 (3.7)
Organ/space	1 (3.7)

^*^ for a history of intestinal carriage with *E. coli* harbouring OXA-48 gene, phenotypically susceptible to meropenem (MIC 1 mg/l)

### Differences in bacterial communities among patients and sample types

There were significant differences of bacterial taxonomic profiles in the analysis with PERMANOVA: postoperative vs preoperative rectal swabs in non-stoma patients (P = 0.005, R^2^ = 0.0435, F = 1.9121; only patients with standard prophylaxis: P = 0.0056, R^2^ = 0.0626, F = 2.1372); preoperative rectal vs intraoperative luminal swabs (P = 0.0145, R^2^ = 0.0317, F = 1.7046); and postoperative rectal vs intraoperative luminal swabs of non-stoma patients (P = 0.0001, R^2^ = 0.0948, F = 4.398; only patients with standard prophylaxis: P = 0.0001, R^2^ = 0.1162, F = 4.2071).

In terms of changes in individual bacterial taxa, we observed large and significant increases in the genus *Enterococcus* and species *E*. *faecalis* when preoperative rectal and intraoperative luminal swabs were compared to postoperative rectal samples (Additional file 1: Table S1). The abundance of unclassified *Blautia* and *Agathobacter rectalis*, both belonging to *Lachnospiraceae*, decreased in postoperative samples. These changes were significant when all patients were considered together or when we analysed only those that received standard prophylaxis. In patients under carbapenem prophylaxis we observed similar changes but with no statistical significance, likely due to the small sample size (n = 5). For example, the median relative abundance of *Enterococcus* in postoperative samples was even higher in patients with carbapenem- than in those with standard prophylaxis (10.3 vs 1.3%).

**Fig. 1:**
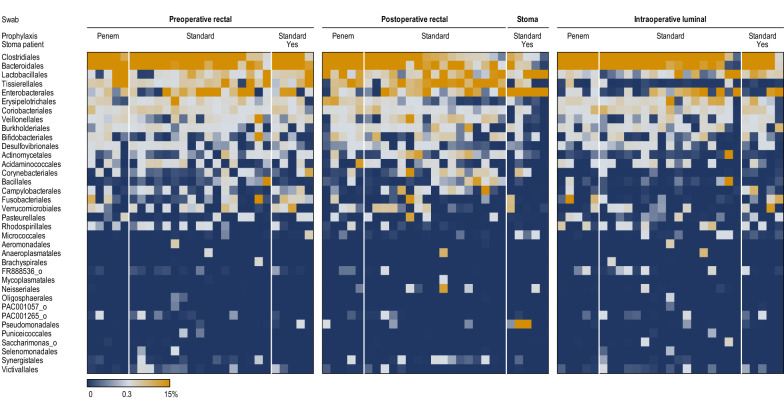
Heat map of the relative abundance of bacterial orders across different sample types and sampling points. The columns correspond to the patients, ordered by decreasing relative abundance of *Clostridiales* within each group

In patients receiving standard prophylaxis, several taxa had higher levels in rectal swabs than in luminal samples. These included anaerobic gram-negative short rod-shaped *Porphyromonas* (phylum *Bacteroidetes*) and anaerobic gram-positive cocci from the *Peptoniphilaceae* family (Additional file 1: Table S1). *Peptoniphilaceae* was the only representative of the order *Tissierellales* (Fig. 1) and the class *Tissierellia* (phylum *Firmicutes*) and was notably represented by the genera *Peptoniphilus*, *Finegoldia*, *Anaerococcus*, *Murdochiella*, *Fenollaria* and *Ezakiella*.

**Fig. 2:**
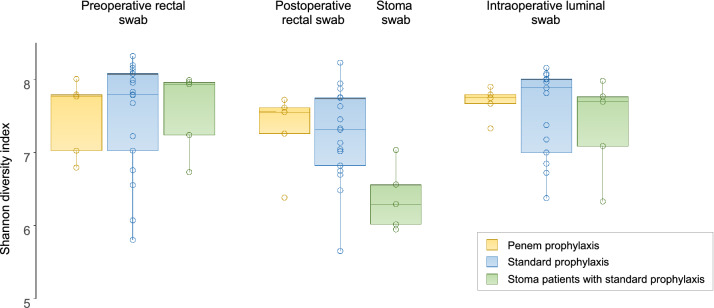
Bacterial diversity across samples from different sites and sampling points. Differences between stoma swabs and all presented groups of luminal or rectal swabs were statistically significant (P < 0.05 by Wilcoxon rank sum test)

**Fig. 3:**
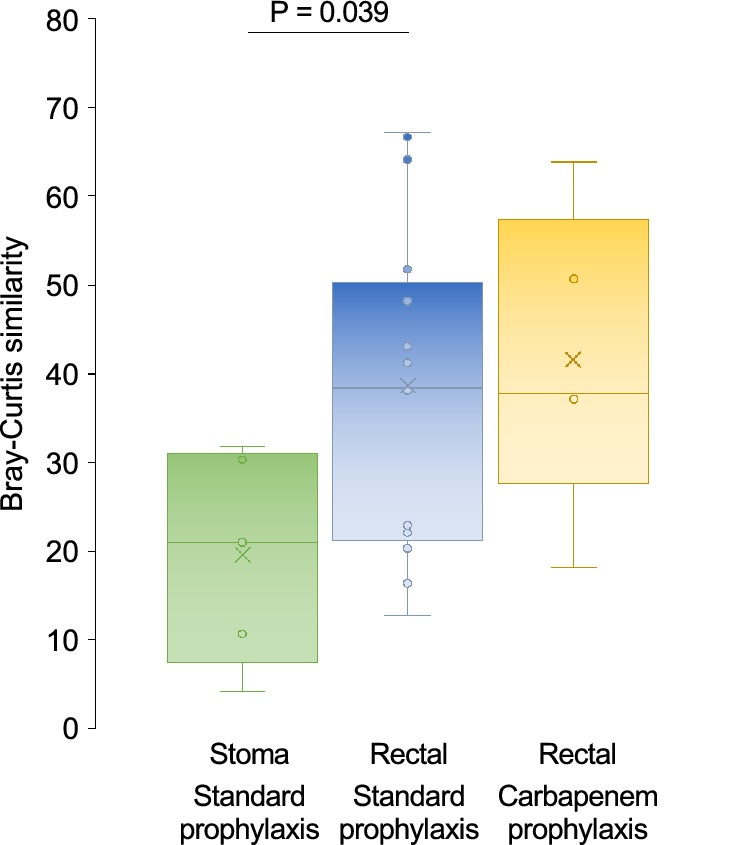
Bray Curtis similarity between preoperative (rectal) and postoperative (stoma or rectal) samples in patients who received cefuroxime + metronidazole (standard) versus carbapenem prophylaxis. P value is indicated only when significant (P < 0.05 by Wilcoxon rank sum test)

In patients receiving standard prophylaxis (all of the 5 stoma patients and 17/22 non-stoma patients), stoma and rectal swab microbiota were significantly different (P = 0.0032, R^2^ = 0.0862, F = 1.8865). Compared to rectal swabs, stoma samples had significantly higher proportion of the order *Enterobacterales* (Fig. 1) and associated higher-level taxa (class *Gammaproteobacteria* and phylum *Proteobacteria*). In addition, the diversity of the stomal microbiota was significantly lower to that or rectal and luminal swabs (Fig. 2) and lower within-patient similarity to preoperative swabs as compared to postoperative rectal swabs (Fig. 3).

**Fig. 4:**
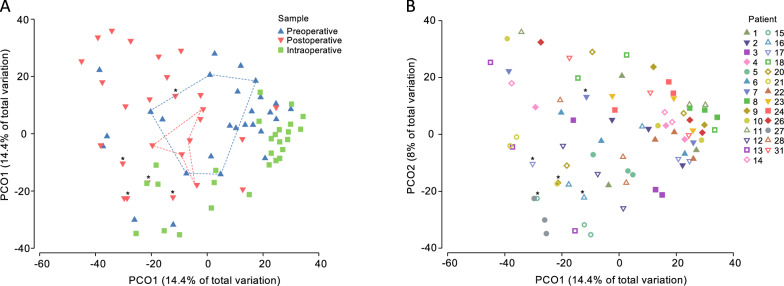
PCoA of beta-diversity of bacterial communities. Each data point on the chart represents a patient-sample combination. **A** Differences between preoperative rectal swabs, intraoperative luminal swab and postoperative (day 3) rectal or stoma (asterisk) swabs. The five pre- or postoperative samples from the subjects who received carbapenem prophylaxis are connected by dashed lines. Other patients (including all stoma patients) received standard prophylaxis (cefuroxime + metronidazole). **B** Differences between patients

Despite all above mentioned variations (Fig. 4A), the samples also clustered by individual (all patients, P = 0.0096, R^2^ = 0.02257, F = 1.8241; non-stoma patients P = 0.0001, R^2^ = 0.07643, F = 2.6068) (Fig. 4B).

### Microbiota differences related to the prophylaxis type

Differences in the postoperative rectal swab microbiota between patients with standard prophylaxis and those with carbapenem administration were significant (PERMANOVA P = 0.03, R^2^ = 0.0711, F = 1.5301), which was not the case when corresponding preoperative swabs were analysed (P = 0.4338). The difference in multivariate dispersion of microbial communities (Fig. 4A) between the two treatments were close to the significance threshold (PERMDISP P = 0.0557) for postoperative but not for the corresponding preoperative samples (PERMDISP P = 0.5629).

Postoperative rectal swabs of patients who received standard prophylaxis had significantly higher relative abundances of the class *Erysipelotrichii* and lower-level taxa belonging to it (order *Erysipelotrichales*, family *Erysipelotrichaceae*, genus *Longicatena* and a not-yet-validated *Longicatena* species JH590969_s) when compared to those with carbapenem, as revealed by ANCOM test.

### Associations between microbiota and patient characteristics

The relationship between patient characteristics and differences in gut microbiome are shown in Table 3. Briefly, although there was a trend towards an association between body mass index and differences in the preoperative gut microbiome, these were not significant.

**Table 3:** Associations between patient and tumour characteristics and overall bacterial profiles of preoperative rectal and intraoperative luminal gut microbiota

Characteristic	P value*	
	Preoperative rectal	Intraoperative luminal
Age	0.1571	0.1869
Female gender	0.9038	0.3199
Body mass index	0.0798	0.1681
Prealbumin	0.4161	0.5159
Glucose	0.6901	0.3293
Preoperative haemoglobin	0.2852	0.1709
Postoperative haemoglobin	0.5422	0.2148
E-ESBL carriage	0.6326	0.7367
Tumour characteristics		
Tumour location (ascending/transverse vs descending/sigmoid)	0.2698	0.0109 (R2 = 0.03425 F = 1.7623)
Tumour stage (T1/T2 vs T3/T4)	0.9298	0.9778
Tumour budding (BD1 vs BD2/BD3)	0.1342	0.2722

^*^based on PERMANOVA or DISTLM tests

There were no significant associations between tumour characteristics and changes in the gut microbiome, except for location of tumour and intraoperative microbiome (Table 3, Additional file 2: Supplementary Fig. 1), which possibly reflects variations in microbiota along the longitudinal gut axis [29]. The microbiota from the distal colon (descending and sigmoid segments considered together), when compared to those from the proximal (ascending plus transverse) part, were significantly depleted in *Aerococcaceae*/*Granulicatella* [median 0.0019, IQR (0.0011–0.0036) vs 0.0356 (0.004–0.2765)] and *Veillonella* [0.0032 (0.0007–0.0207) vs 0.0559 (0.018–0.3472)], while being enriched in *Christensenellaceae* [(1.4842 (0.6272–2.3287) vs 0.0987 (0.0016–0.2384)].

There was no significant association between E-ESBL carriage and differences in gut microbiome in any of the samples.

## Discussion

This prospective hypothesis-generating pilot study evaluated changes in the rectal microbiota of colon cancer patients requiring a surgical intervention. The results suggest that surgery, including perioperative antimicrobial prophylaxis, induces rapid changes in the gut microbiota.

There is a dearth of studies evaluating perioperative changes in the rectal microbiome of colon cancer surgery patients. A prospective study on 54 US patients showed that both preoperative and postoperative composition of the rectal microbiome, in particular higher proportions of *Proteobacteria* and *Bacteroidetes* and lower levels of *Actinobacteria* and *Firmicutes,* were associated with postoperative ileus, but not SSI [30]. These results are not directly comparable to ours for several reasons: first, Shogan et al. [12] included both colon and rectal surgery patients. Second, they included patients with diverse surgical indications: 61% were operated for non-cancer related diseases such as inflammatory bowel disease, which may be associated both with altered rectal microbiome and development of postoperative complications. This is reflected also by the fact that 14% patients experienced postoperative ileus. Third, 82% of their patients underwent mechanical bowel preparation, which is also known to alter the microbiome. In contrast, our study was a more homogenous population, with minimal disruption of the microbiome apart from surgery and perioperative antimicrobial prophylaxis.

Van Praagh et al. published a cohort study evaluating the association between the local mucosal tissue microbiome at the site of anastomosis with the risk of anastomotic leakage of 123 colorectal surgery patients in the Netherlands, most of whom (95%) were operated for cancer [31]. Anastomotic leakage was associated with increased relative abundance of *Lachnospiraceae* and *Bacteroidaceae*. Again, patients in their cohort underwent mechanical bowel preparation [32], and thus these results do not reflect the “native” microbiome.

The study by Jin et al. assessed the ability of the intraoperative tissue microbiome composition to predict postoperative ileus in colorectal cancer patients in China [33]. Their results suggest decreased relative abundance of *Faecalibacterium* in patients who would later develop postoperative ileus, consistent with the results by Shogan et al. [30].

Another observational study based on targeted PCR assays to evaluate changes in bacterial counts before and after colon surgery, showed decreases in beneficial obligate anaerobes and increases in pathogenic bacteria; this study did not have the potential to provide a comprehensive overview of the microbiome because it did not use next-generation sequencing-based techniques [34].

Several interventional human studies suggest that preoperative administration of probiotics or synbiotics to colorectal surgery patients may be associated with postoperative complications, including infection; these studies, however, are fraught with methodological shortcomings, and the level of uncertainty on the effectiveness of this intervention [35]. One study using 16S rRNA gene-based metataxonomics was a single-centre randomized clinical trial which showed that perioperative administration of probiotics (*Bifidobacterium longum*) in patients undergoing colorectal surgery increases the proportions of *Actinobacteria*; there were, however, no statistically significant differences between the intervention group and control group either in terms of composition of the microbiota nor in terms of clinical outcome, probably due to the small size of the study [36].

Animal models show that colon surgery and perioperative measures (perioperative antibiotic prophylaxis, fasting, mechanical bowel preparation) alter the microbiome [12]. In rats, colectomy is associated with important increases in the amount of pathogenic bacteria (*Enterococcus*, *Escherichia*) [37].

Strengths of our study include state-of-the-art next-generation sequencing and bioinformatics techniques, a well-defined homogenous study population, as well as robust statistical analyses. Ours was a prospective study, decreasing the risk of bias associated with retrospective studies.

The main limitation of this study was the sample size, which was small, as was the number of events we had initially planned to evaluate (SSI). Despite this, we present interesting and novel findings, which will need to be confirmed in larger studies. Another limitation is the short duration of follow-up of the rectal/gut microbiome; indeed, it would have been interesting to study the dynamics of recovery of the different taxa, and investigate factors associated with this recovery. Finally, we only focused on the intestinal microbiome, whereas a more comprehensive approach also including the skin and nares microbiome should be adopted since the members of these communities can cause SSI [38, 39].

Understanding changes in the gut microbiome of colon cancer surgery patients is important, yet understudied [40]. Further, well-designed prospective studies with homogenous populations should be performed, in combination with other explanatory factors in order to obtain significant insights [41]. The research agenda should also include the changes of the gut microbiome specifically associated with mechanical bowel preparation and, more importantly, non-absorbable oral antimicrobials [42].

## Conclusions

In conclusion, our results showed rapid changes in the rectal microbiota following colon surgery. We also observed differences in microbiota structure between stoma and rectal swabs, whereas previous studies showed good similarity between rectal swab and stool microbiota [43, 44]. Our findings highlight that results obtained by using rectal swab (and possibly faecal matter) microbiota, as a proxy for colonic microbiota, should be interpreted with caution due to the differences between rectal and luminal colonic bacterial communities, notably in the relative abundance of anaerobic gram-positive cocci and gram-negative rods.

## Availability of data

Sequencing data were submitted to the European Nucleotide Archive (ENA; www.ebi.ac.uk/ena; study number: PRJEB44214).

## Supplementary Information

**Additional file 1: Table S1.** Heat map showing only bacterial taxa where significant changes in relative abundance were present in at least one of the pairwise comparisons.

**Additional file 2: Figure S1.** PCoA showing similarities/differences between bacterial communities from different colonic segments. In all pairwise comparisons between the four groups, only differences between ascending vs sigmoid colonic communities were statistically significant (PERMANOVA P = 0.0449, R^2^ = 0.0855, F = 1.4952).
