# Supplementary figures and images for: Changes in the gut bacterial communities in colon cancer surgery patients: an observational study
Source: Gut Pathog. 2022 Jan 4;14:2. doi: 10.1186/s13099-021-00477-7 (PMC8729125; doi:10.1186/s13099-021-00477-7)

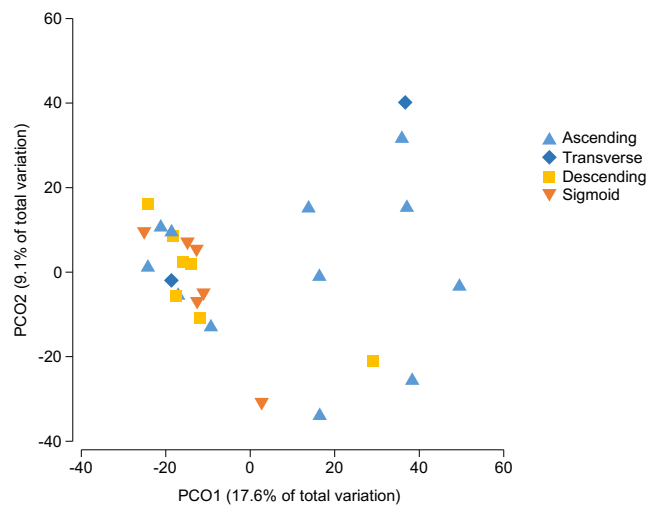

Supplement: Supplementary file 2 — Additional file 2: Figure S1. PCoA showing similarities/differences between bacterial communities from different colonic segments. In all pairwise comparisons between the four groups, only differences between ascending vs sigmoid colonic communities were statistically significant (PERMANOVA P = 0.0449, R2 = 0.0855, F = 1.4952). [file 13099_2021_477_MOESM2_ESM.pdf]
